# The Patient and Public Involvement Activities of the COMET Initiative

**DOI:** 10.1186/1745-6215-16-S3-P7

**Published:** 2015-11-24

**Authors:** Heather Bagley, Bridget Young, Paula R Williamson

**Affiliations:** 1 Department of Biostatistics, University of Liverpool, Liverpool, UK; 2 Institue of Psychology Health and Society, University of Liverpool, Liverpool, UK

## Background

The COMET Initiative recognises the expertise and crucial contribution of patients and carers in developing core outcome sets and research more generally. Core outcome sets need to include outcomes that are most relevant to patients and carers, and the best way to do this is to involve patients and carers in their development. COMET is developing resources to help patients and carers to get involved in this work.

## Method

In March 2014 COMET hosted a collaborative meeting between core outcome set developers, UK public involvement organisations and the COMET Initiative. The aims of the meeting were to: Raise awareness amongst attending public involvement organisations about the work of COMET; identify resources that are relevant to facilitate public involvement in the work of COMET and discuss a strategy for engaging patient organisations in the work of COMET. The COMET initiative is also developing plain language resources to support patient and public involvement in COS studies. It is also piloting a tool to help involve patients in the design of a COS study.

## Results

As a result of the Involving People event COMET has developed an initial Patient and Public Involvement strategy and is establishing a People and Patient Participation, Involvement and Engagement (PoPPIE) working group to take forward the strategy and action plan. Two plain language resources have so far been developed with the involvement of patients and the public, these include a summary of what core outcome sets are and the work of the COMET Initiative and a description of what a Delphi process is. Piloting is underway for the tool to support researchers in designing their core outcome set study with the involvement of patients. COMET worked collaboratively with EURO-DIS (European rare disease patient organisation) and presented a webinar about COS and COMET.

